# Prevalence and correlates of gender inequitable norms among young, church-going women and men in Kinshasa, Democratic Republic of Congo

**DOI:** 10.1186/s12889-018-5742-9

**Published:** 2018-07-17

**Authors:** Hendrew Lusey, Miguel San Sebastian, Monica Christianson, Kerstin E. Edin

**Affiliations:** 1 World Council of Churches, Ecumenical HIV and AIDS Initiative and Advocacy (EHAIA), Central Africa regional office, C/o Salvation Army Headquarters, Avenue colonel Ebeya No. 23, B.P: 8636, Kinshasa Gombe, Democratic Republic of Congo; 2 Epidemiology and Global Health, Department of Public Health and Clinical Medicine, Umeå University, Umeå, Sweden; 3 Sexual and Reproductive Health, Department of Nursing, Umeå University, Umeå, Sweden

**Keywords:** Gender inequitable norms, Gender equity, Church youths, Masculinities, Cross-sectional survey, DR Congo

## Abstract

**Background:**

Prolonged political instability may have exacerbated gender inequitable beliefs in the Democratic Republic of Congo (DRC). The aim of this study was to assess attitudes related to gender-equitable norms and its determinants among young, church-going women and men in Kinshasa, DRC.

**Method:**

Data were collected through a cross-sectional survey with 291 church-going women and 289 men aged 18–24 years old, residing in three disadvantaged communes of Kinshasa. Variables included sociodemographic characteristics, attitudes towards gender equality, and responses to issues related to the gender-equitable men (GEM) scale. The GEM scale is a 24 item-questionnaire developed to measure attitudes towards gender equitable norms. Logistic regression was applied to discover the associations between the independent variables and the GEM outcome.

**Results:**

Our study reflected the existence of attitudes hampering gender equality that were endorsed by both women and men. For example, 91.4% of women and 83% of men agreed with the statement “a woman’s most important role is to take care of her home and cook for her family”. Similarly, 88.3% of women and 82.9% of men concurred with the idea that men need more sex than women. These findings coexisted with a few equitable norms, because 93.7% of women and 92.3% of men agreed that a man and a woman should decide together if they want to have children. A positive association was found in both women and men between being educated, being single and separated and having supportive attitudes towards gender equality and a higher GEM scale score. Residency in Camp Luka and Masina was also a significant social determinant associated with equitable gender norms among men whilst job status was only significant among women.

**Conclusion:**

While both women and men had high levels of gender inequitable norms, those with more education, single, and with supportive attitudes to gender equality had high GEM scale scores. The results highlight an urgent need for the church to challenge and change gender norms among church youths.

## Background

The United Nations has prioritized the achievement of gender equality and women’s empowerment as one of the main ways of ensuring the sustainable development goals [1]. The latest United Nations’ Development Programme report stated that the gender equality index in sub-Saharan Africa (SSA) was low [2], potentially heightening unequal gender norms [3]. Both women and men receive societal messaging in early life and internalize unequal gender norms that dictate how women and men are supposed to behave [4]. For instance, women may have limited abilities to negotiate safer sex with male partners [5]. This attitude may be supported by a belief that considers women who carry condoms in their bags as ‘promiscuous’, pitting pregnancy and HIV prevention against social standards of women and sexuality [6–8].

Conversely, having unprotected sex with several partners and using violence against women might be common expectations for men, suggesting that men may drive HIV in sub-Saharan Africa [9, 10]. Despite a growing recognition that changing inequitable gender norms is key to successful HIV prevention, most studies are mainly focused on women, perceived as facing negative consequences to their health as a result of socio-economic and cultural disadvantages [10, 11]. However, research reveals that gender equality is not possible without a meaningful men’s engagement as partners in the processes and African countries that seriously seek to engage men in challenging social norms in relation to gender inequality tend to perform better in health [12]. For instance, a South African study examined how men responded to change towards gender equality and found that positive changes in gender beliefs and practices amongst men were correlated with improved health outcomes, such as increased condom use and HIV testing [10]. These results are consistent with studies arguing that gender transformative programmes can engage men as change agents [13].

Despite some ambivalence and resistance [9], research suggests that African men can learn to be positive about gender equality and actively support it in family and community life [14–16]. Research has also shown that integrating women and men as active partners in health interventions could be a useful strategy to transform gender inequalities [16, 17]. In solidarity with women, men can be mobilized to endorse gender equality and this might be based on their respective desire for mutual respect and healthy lives [18]. Studies that examined gender equitable men (GEM) domains found evidence supporting that gender equitable attitudes can be a protective factor against sexually transmitted infections (STIs) and HIV. For instance, men with equitable attitudes were more likely to report healthy intimate relationships in which they discuss and use condom with partners [19], suggesting the importance to focus interventions already in young age to promote gender equity [20].

Churches are an integral part of social life in many African societies where they have wide networks and provide nearly 70% of health services and mainly to the most marginalized people [21]. Churches also promote moral norms among youths and may have unparalleled advantages over other sectors on addressing inequitable attitudes that may start in adolescence and may continue in adulthood in the absence of spaces for critically reflection on gender [22]. Specifically, churches may challenge and change norms of gender inequality informed by the church beliefs, teaching and practices in deprived urban areas where church leaders may continue to be seen as trusted figures among church youths [23].

For instance, a study conducted in Zambia showed the way some men exhibited harmful norms of masculinities of having sex with multiple partners, perpetrating violence against partners and drinking heavily [24]. When they were taught Christian norms of sexuality, 20% of men in the study population adopted new patterns of behaviours in becoming faithful, sticking to their partners and stopping abusive alcohol consumption [24]. Research indicates that Bible studies may have opened up safe spaces where hegemonic norms of masculinities were discussed and challenged and alternative masculinities were suggested [25, 26]. In spite of this, gender inequality remains deeply entrenched in many African churches where scriptures may be used to justify men as the dominant partners and women as subservient to the desire of men [24]. Hence engaging churches to address gender inequality may entail challenges, but with careful alignment as well as concerted actions, much can be gained.

While DRC is a country with vast natural resources, most Congolese live in abject poverty attributable to political instability, resource mismanagement, and armed conflict. DRC ranked both at the bottom of the Gender Equality Index (144th out of 148 countries) (Davis L, Fabbri P, Muthaka AI. Democratic Republic of Congo – DRC: Gender Country Profile. Commissionned by the Swedish embassy in collaboration with DFID, the European Union Delegation and the Embassy of Canada in Kinshasa, Unpublished) and Human Development Index (176th out of 188) [27]. DRC did not achieve the Third Millennium Development Goal regarding gender equality (Rapport national Objectifs Millénnaires du Développement (OMD): Evaluation des progrès accomplis par la République Démocratique du Congo dans la réalisation des objectifs du millénaire en 2012, unpublished), making DRC one of the world’s most challenging countries for women to live in [28]. The International Men and Gender Equality Survey (IMAGES) conducted a study in DRC in 2011, with participants 18–59 years old. It found that both Congolese women and men endorsed inequitable gender norms, viewing women as responsible for completing household chores and men as heads of the households [28].

The distribution of the population according to the religious affiliations in DRC is: Catholic (31%); Protestant (30%); other Christian Churches (34%); indigenous religions (3%); and Muslims (2%) [29]. The Ecumenical HIV and AIDS Initiative and Advocacy (EHAIA) have sought to equip Congolese churches with support to promote equitable relationships but research on gender norms among church goers is lacking in DRC [30]. In an attempt to fill parts of this knowledge gap, our study aimed at assessing attitudes related to gender equitable norms and its determinants among young, church-going women and men in Kinshasa, DRC.

## Methods

### Study design and setting

This cross-sectional survey was carried out in Bumbu, Camp Luka, and Masina, three deprived peri-urban communes located in Kinshasa, the capital of DRC. The study sites were purposively selected given the youth work that the Église du Christ au Congo and Salvation Army was undertaking in these areas. In addition, the first author had collaborated with the leadership of these churches through his engagement with EHAIA [31].

### Recruitment and data collection

The first author acquired the French translated questionnaire IMAGES from Promundo, a nongovernmental organisation founded in Brazil, which promotes equitable masculinities and equitable gender relations locally and globally [32]. We pretested the questionnaire among ten young women and ten young men from churches located in deprived communes of Kinshasa outside the study sites. The pre-test enabled the team of researchers to find out if the survey statements were culturally appropriate, understandable and clear for the local church youth context. Overall, the questionnaire was apposite to participants, except for a few items that the research team reworded to facilitate the respondents’ understanding. For example, one item was initially labelled as follows: “work to achieve gender equality today benefit mostly well-to-do people”. We changed the term “well-to-do-people” with “wealthy people”.

The local parish pastors recruited eligible participants for this study based on the following criteria: to be young women and men (18–24 years old), belonging to the Salvation Army and the Église du Christ au Congo, living in the three selected communes during the time data was collected, able to read and write in French, and volunteered to be involved in the study. We decided to interview this target group because young people aged 18 years do not need parental permission for participating in research and those aged 24 years are still considered as youths according to the World Health Organisation definition [33]*.*

The authorisation to carry out this study was provided by the leadership of both churches involved and the first author made contacts with the respective pastors who recruited the respondents at the congregational levels, mainly on Sundays. Once the church youths had gathered to take part in the study, the first author provided them with a self-administered questionnaire. The participants read and filled in the questionnaire autonomously within the relatively quiet church premises. In some instances, particularly for respondents who found certain statements difficult to grasp, the first author read the questions aloud to enable everyone to complete the survey. In general, it took approximately one hour for each participant to fill in the questionnaire.

The study was carried out from March to April, 2016 and in total 750 church-going youths were invited to participate. However, 88 participants were excluded for not meeting the inclusion criteria (being outside the appropriate age range, residing outside the study settings when data were collected, and belonging to a church other than the two selected churches): 67 did not turn up, and 15 declined the invitation for personal reasons. Finally, 580 were included, of whom 291 women and 289 men completed the questionnaires [31].

### Variables

Our questionnaire is an adapted version of the International Men and Gender Equitable Survey (IMAGES) that was organised in three groups: *sociodemographic characteristics*, *attitudes towards gender equality*, and the *Gender Equitable Men scale* (GEM) [34]. In addition to the more general sociodemographic characteristics, our version of the questionnaire included a few specific items related to the targeted-church aspects of the study, such as the church membership and attendance.

#### Sociodemographic characteristics

Although gender was dichotomised into women and men [35], age was categorised into three groups (18–19, 20–21, and 22–24 years old). The civil status included single, married/cohabiting, or separated. Education had three categories, primary, secondary, and university levels. We also collected some information regarding the respondents’ residences and church affiliations. Although the variable main source of income included the head of their households (parents) or the respondents themselves, the labour status was categorised as currently employed, student, and unemployed. Each participant ticked only one option.

#### Attitudes towards gender equality

This part of the original IMAGES questionnaire comprises several themes, notably men and women’s practices, attitudes related to gender norms, gender equality, household dynamics and the men’s involvement as fathers, intimate partner violence, health, and stress [28]. After we had piloted the original IMAGES questionnaire, we identified the most relevant items for the target group of young people. We shortened the GEM scale to assess items that were more specifically related to our study aim of assessing gender equality among young people. As an example, we did not include factors about parenting because most of our participants were unmarried youths. In both South Sudan and Uganda, they also adapted the IMAGES questionnaire by taking out certain statements considered as less appropriate for their studies [36, 37]*.* Our survey included 16 statements and responses fell into four categories as participants were asked to ascertain if they (1) completely or (2) partly agreed or they (3) partly or (4) completely disagreed with the statements. An index, created by the sum of all of the items, was developed and divided into terciles representing low, medium, and high levels of attitudes towards gender equality. Responses by young, church-going women and men to the questions regarding attitudes are reported in Appendix. Higher scores on the index denote greater gender equitable attitudes.

#### The GEM scale

The GEM scale was originally developed in low income settings in Brazil and used as a tool to measure changes in gender-related interventions [32, 38, 39]. The GEM scale has been used and validated in more than 20 countries around the world [40] including Brazil, India and Kenya [6], South Sudan [36], and in DRC [14, 28]. In the same vein, GEM has been used among women to examine gender norms in two earlier studies: in South Sudan [36] and DRC [28]*.* Initially, the GEM scale comprises 24 items related to gender and domestic chores, violence, sexual relationships, masculinities, and sexual and reproductive health. While our study included a list of 16 statements, we excluded the eight remaining GEM items after reflecting on the misunderstandings faced by young people involved in the pilot study [31]. A similar approach as ours, with regard to the GEM scale, was used in South Sudan and in DRC [28, 31, 36]. We summed up and grouped the items into terciles for descriptive purposes and later on, we dichotomised them using the mean as the cut-off point in low and high GEM scale for the regression analysis, representing low and high support for gender equitable norms [31, 41]. The Cronbach alpha regarding the GEM for women and men were 0.700 and 0.738 respectively, indicating an acceptable consistency of the scale [42].

### Data analysis

As mentioned at the outset of this paper, the respondents’ survey responses were written on the questionnaire format that the first author entered into an Excel sheet and transferred later on the whole data file to STATA 13.1 for statistical analysis. Because the study was looking for differences between women and men, all analyses were stratified by gender. First of all, the percentages of responses related to sociodemographic characteristics of young women and men, attitudes towards gender equality, and the GEM scale were calculated. Statistical gender differences for the various domains of the GEM scale were assessed using the chi-squared test. In the descriptive part, we used terciles as to do so is part of the GEM guidelines [32]. But we divided them into two groups according to the mean in order to do the logistic regression. In a second step, a logistic regression analysis was performed to find out associations between the sociodemographic characteristics and gender-equality attitudes of the participants with the GEM scale. Additionally, we calculated crude and adjusted odds ratios (OR) and their 95% confidence intervals (CI). Significant variables (*p* < 0.05) in the crude model were included in the adjusted models. Once the logistic regressions were established, the goodness-of-fit of the models was calculated using the Hosmer Lemeshow’s goodness-of-fit test to estimate that the presumed models were appropriately specified in the third step. Finally, given the relative small sample, predictable variables were organized in five groups. *P*-values were not significant, indicating a good model fit.

### Ethical approval

This study was approved in 2010 by the institutional review board of the School of Public Health at the University of Kinshasa, DRC. The study’s aim and purpose were explained to the women and men taking part, who were ensured anonymity. Written informed consent was obtained from all study participants.

## Results

Table 1 presents the sociodemographic characteristics of 580 participants, where 29.5% of women and 55.3% of men were 18–19 years old. Nearly half (49.4%) of the women and 65.4% of the men were single. About 16.8% of women and 9.3% of men surveyed had finished primary school. Similarly, 46.3% of women and 33.6% of men held a secondary school level. Finally, 37.7 and 29.8% of women and men surveyed, respectively, had a university degree. Most of the participants (87.9% of women and 91.7% of men) belonged to the Salvation Army and the rest belonged to the Église du Christ au Congo. In the group of participants, 87.6% of women and 77.1% of men reported that they attended the church services regularly. Most participants (65.9% of women and 75% of men) reported the head of their household (their parents) as the main source of their income. Almost one-third (29.5%) of women and close to one-fourth (22.4%) of men stated that they were employed. Around one-fifth of both women (17.8%) and men (20%) scored low in the gender equitable attitudes questionnaire, though a larger percentage of women scored higher than men (47.2% versus 17.9%).

**Table 1:** Sociodemographic characteristics, attitudes towards gender equality, and Gender Equitable Men Scale Scores of young, church-going women and men in Kinshasa, Democratic Republic of Congo

	Young women	Young men
	*N* (%)	*N* (%)
Age groups		
18–20	86 (29.5)	160 (55.3)
21–22	69 (23.7)	67 (23.1)
23–24	136 (46.7)	62 (21.4)
Civil status		
Married/cohabiting	85 (29.2)	64 (22.1)
Single	144 (49.4)	189 (65.4)
Separated	62 (21.3)	36 (12.4)
Highest grade		
Primary school	46 (16.8)	27 (9.3)
Secondary school	155 (49.4)	163 (56.4)
University	89 (30.6)	99 (34.2)
Place of residence		
Bumbu	46 (15.9)	105 (36.4)
Masina	134 (46.3)	97 (33.6)
Camp Luka	109 (37.7)	86 (29.8)
Church belonging		
Salvation Army	256 (87.9)	265 (91.7)
Église du Christ au Congo	35 (12)	24 (8.3)
Main source of income		
Respondents themselves	99 (34)	72 (24.9)
Heads of households	192 (65.9)	217 (75)
Labour status		
Unemployed	103 (35.4)	133 (46)
Students	100 (34.3)	91 (31.4)
Employed	86 (30.2)	65 (22.4)
Attitudes to gender equality		
Low	50 (17.1)	58 (20)
Medium	121 (41.5)	179 (61.9)
High	120 (41.2)	52 (17.9)
GEM		
Low	162 (55.6)	112 (38.7)
Moderate	122 (41.9)	87 (30.1)
High	7 (4.2)	90 (31.3)

The next section summarises the views expressed by the participants by domains of the GEM scale (Table 2). More women (55.6%) than men (38.7%) had low scores on the GEM scale. Overall, the GEM domains related to gender and domestic chores, violence and sexual relationships were statistically significant among both women and men, whereas domains related to masculinities, sexual and reproductive health were not statistically significant. For example, 71.4% of women and 66% of men suggested that a woman’s most important role is to care for her home and cook for her family. Additionally, approximately half of women and men agreed that, to be a man, a man needs to be tough. In the same vein, 83.4% of women and 82.7% of men concurred with the idea that they would be outraged if their partners asked them to use a condom. The views about the GEM domains related to violence and sexual relationships, however, differed more between women and men. For instance, 70.7% of women vs. 50.8% of men disagreed that a woman should tolerate violence in order to keep the family together. The majority of the women (68.3%) and half (50.8%) of the men agreed that men need more sex than women do.

**Table 2:** Scores of the Gender-Equitable Men Scale for young, church-going women and men

		Young women	Young men	P-value
Survey statements	Responses	*N* (%)	*N* (%)	
Gender and domestic chores				
A woman’s most important role is to take care of her home and cook for her family	Totally agree	208 (71.4)	191 (66)	
	Partially agree	58 (19.9)	49 (16.9)	
	Disagree	25 (8.5)	49 (16.9)	0.01
Changing diapers, giving a bath, and feeding kids is the mother’s responsibility	Totally agree	186 (63.9)	185 (64.)	
	Partially agree	85 (29.2)	53 (18.3)	
	Disagree	20 (6.8)	51 (17.6)	< 0.05
A man should have the final word about decisions in his home	Totally agree	206 (70.7)	242 (83.7)	
	Partially agree	58 (19.9)	29 (10)	
	Disagree	27 (9.2)	18 (6.2)	< 0.05
Violence against women				
A woman should tolerate violence in order to keep the family together	Totally agree	48 (16.4)	109 (37.5)	
	Partially agree	37 (12.7)	60 (20.7)	
	Disagree	206 (70.7)	147 (50.8)	< 0.05
There are times when a woman deserves to be beaten	Totally agree	79 (27.1)	147 (50.8)	
	Partially agree	122 (41.9)	85 (29.4)	
	Disagree	90 (30.3)	57 (19.7)	< 0.05
Sexual relationships				
Men need more sex than women do	Totally agree	199 (68.3)	147 (50.8)	
	Partially agree	60 (20.6)	93 (32.1)	
	Disagree	32 (11)	49 (19.8)	< 0.05
Men are always ready to have sex	Totally agree	175 (60.1)	131 (45.3)	
	Partially agree	64 (21.9)	96 (33.2)	
	Disagree	52 (17.8)	62 (21.4)	< 0.05
Masculinities				
To be a man, you need to be tough	Totally agree	154 (52.9)	145 (50.1)	
	Partially agree	80 (27.4)	85 (29.4)	
	Disagree	57 (19.5)	59 (20.4)	0.79
If someone insults me, I will defend my reputation, with force if I have to	Totally agree	182 (62.5)	116 (40.2)	
	Partially agree	47 (16.1)	91 (31.6)	
	Disagree	62 (21.3)	82 (28.1)	< 0.05
I would never have a gay friend	Totally agree	187 (64.2)	166 (57.4)	
	Partially agree	34 (11.6)	53 (18.3)	
	Disagree	70 (24.6)	70 (24.2)	0.06
Sexual and reproductive health				
I would be outraged if my partner asked me to use a condom	Totally agree	156 (50.6)	137 (47.4)	
	Partially disagree	62 (21.3)	87 (30.1)	
	Disagree	73 (25)	65 (22.4)	< 0.05
A man and a woman should decide together if they want to have children	Totally agree	244 (83.8)	239 (82.7)	
	Partially agree	26 (8.9)	28 (9.6)	
	Disagree	21 (7.2)	22 (7.6)	0.93
It is a woman’s responsibility to avoid getting pregnant	Totally agree	128 (43.9)	119 (49.1)	
	Partially agree	128 (43.9)	87 (30.1)	
	Disagree	35 (12)	83 (28.7)	< 0.05
Men don’t talk about sex, they just do it	Totally agree	170 (58.4)	119 (41.1)	
	Partially agree	57 (19.5)	87 (30.1)	
	Disagree	64 (21.9)	83 (28.7)	< 0.05
Men should be embarrassed if they are unable to get an erection	Totally agree	119 (40.8)	153 (52.9)	
	Partially agree	96 (32.9)	68 (23.5)	
	Disagree	76 (26.1)	68 (23.5)	< 0.05

The results of the logistic regression of factors associated with high GEM scale scores among young, church-going women and men is presented in Table 3. In the crude analysis, several factors such as being educated, being single and separated, place of residence and having supportive attitudes towards gender equality were significantly related to a higher GEM scale score for both women and men. However, job status and source of income were only significantly associated with a high GEM scale score among women. In the multivariate regression model, young, church-going men but not women who lived in Camp Luka and Masina had greater odds of having high GEM scores. For education, a higher GEM scale score was found among young, church-going women and men (although not significant for the latter) with secondary education and higher, compared with those having a primary level education. In addition, being a student as well as being unemployed were found to be positively associated with a high GEM scale score only among women. A strong association between attitudes to gender equality and the GEM scale score was found in both women and men. For instance, women with medium equity (AOR = 8.67; 95% CI = 3.60–20.89) and high equity (AOR = 4.00; 95% CI = 1.63–9.81) supported attitudes towards gender equality. This was similar for men with medium equity (AOR = 2.56; 95% CI = 1.30–5.04) and those with high equity (AOR = 7.09; 95% CI = 2.81–17.89).

**Table 3:** Factors related to a high Gender-Equitable Men Scale reported by young, church-going women and men: logistic regression analysis with crude and adjusted odds ratios (OR) and their 95% confidence intervals (CI)

	Women		Men	
Factors	Crude OR (95% CI)	Adjusted OR (95% CI)	Crude OR (95% CI)	Adjusted OR (95% CI)
Age				
23–24	1	–	1	–
21–22	1.13 (0.63–2.02)	–	0.56 (0.28–1.12)	–
18–20	1.26 (0.73–2.17)	–	1.02 (0.57–1.84)	–
Education				
Primary school	1	–	1	1
Secondary school	1.61 (0.80–3.23)	1.35 (0.60–3.00)	3.04 (1.22–7.58)	2.65 (0.99–7.11)
University	2.21 (1.05–4.65)	3.14 (1.28–7.67)	3.43 (1.33–8.84)	2.54 (0.90–7.16)
Place of residence				
Bumbu	1	1	1	1
Camp Luka	0.34 (0.17–0.70)	0.95 (0.44–2.05)	2.53 (1.41–4.56)	2.27 (1.18–4.38)
Masina	0.79 (0.40–1.55)	0.53 (0.23–1.21)	3.10 (1.75–5.52)	3.29 (1.73–6.25)
Civil status				
Married	1	1	1	1
Single	2.15 (1.23–3.75)	2.15 (1.11–4.17)	2.75 (1.51–5.02)	3.20 (1.64–6.25)
Separated	1.21 (0.61–2.40)	1.93 (0.83–4.50)	2.75 (1.18–6.39)	3.54 (1.36–9.21)
Job status				
Employed	1	1	1	–
Students	0.52 (0.34–0.71)	2.69 (1.44–5.01)	0.89 (0.68–1.16)	–
Unemployed	2.69 (1.44–5.01)	1.57 (1.05–2.34)	1.05 (0.78–1.40)	
Church belonging				
Salvation Army	1	–	1	**–**
Église du Christ au Congo	0.53 (0.25–1.14)	–	0.69 (0.30–1.60)	**–**
Main sources of income				
Themselves	1	–	1	–
Heads of household	2.00 (1.20–3.31)	1.23 (0.61–2.45)	1.46 (0.85–2.50)	–
Attitudes to gender equality				
Low equity	1	1	1	1
Medium equity	6.75 (3.08–14.80)	8.67 (3.60–20.89)	2.00 (1.07–3.76)	2.56 (1.30–5.04)
High equity	2.23 (1.01–4.90)	4.00 (1.63–9.81)	9.33 (3.84–22.63)	7.09 (2.81–17.89)

## Discussion

Our study provides evidence of attitudes and beliefs that may hamper gender equality and demonstrates that women and men in this study endorsed inequitable gender norms. Nonetheless, a positive association was found for both women and men between being educated, being single and separated, and having supportive attitudes towards gender equality and higher GEM scale scores.

### GEM domains

Overall, our findings revealed similarly poor gender-equity scores for both women and men for gender and domestic chores, masculinities, and sexual and reproductive health; these findings are in line with the DRC-IMAGES study [28]. For instance, our study indicated that most respondents considered men as heads of the household and women as caregivers. This may suggest a belief among men and women that women should accept subservient positions. Research has reported that some men and women in DRC tend to consider women as personal property whose job it is to carry out domestic chores after payment of the bride price [43]. Our findings about gender norms related to masculinity were also consistent with the DRC-IMAGES study, which stated that both Congolese women and men have internalised attitudes that support men’s toughness [28]. Research revealed that these dominant forms of masculinities not only have a negative impact on girls and women but also undermine the health and well-being of boys and men [44]. Respondents largely reported agreement with statements of inequitable norms regarding sexual and reproductive health, which hold women as being primarily responsible for preventing pregnancy. However, these attitudes coexisted with a few positive gender-equitable norms. For example, almost all women and men (92.7 and 91.3%, respectively) in our study, compared with 45.6 and 53.7% of women and men, respectively, in the DRC-IMAGES study, concurred with the idea that a man and a woman should decide together if they want to have children [28].

By contrast, respondents’ views about violence against women and sexual relationships were sharply different among women and men. In this regard, the DRC-IMAGES study [28] and our study confirmed that more men (79.2%) than women (69%) agreed with the statement “there are times when a woman deserves to be beaten”. It is worthwhile noting that not all of the results related to gender norms were inequitable. For instance, the statement “a woman should tolerate violence in order to keep the family together” met with disagreement from 70.7% of women and 50.8% of men in our study, compared with 45.1% of women and 34.3% of men in a recent Congolese study [43]. In the GEM-related domain of sexual relationships, 88.3% of women (our study) versus 89.6% (DRC-IMAGES study) and 82.9% of men (our study) versus 70.5% (DRC-IMAGES study) affirmed that men need more sex than women [28].

Several issues might explain some of the differences between the DRC-IMAGES study [28] and ours. While the DRC-IMAGES study was carried out in North-Kivu in the eastern part of the country, which has faced and is still facing violent armed conflicts, our study was conducted in Kinshasa, the western part of DRC, which in comparison seems to be quite “peaceful” despite all its riots. The target group for the DRC-IMAGES study was people aged 18–59 whereas in our study, we include the perspectives of church youths aged 18–24. Our study focuses on young church goers drawn from deprived urban areas compared to the general population particularly those who lived in rural areas outside Goma and in military bases near Goma as internal displaced people who were the target groups in the DRC-IMAGES study.

In comparison to the DRC-IMAGES study, our church-going youths mainly scored gender inequitable statements for several reasons. The prevailing notions of gender in DRC are largely based on norms rooted in unequal power relations characterized by violence experienced by women and men’s domination in decision making process [28]. In addition, gender socialisation of both women and men may begin early in life in DRC and their behaviours may be rehearsed and carried out into adulthood particularly in deprived areas where youths may have limited opportunities for adhering to alternative masculinities and gender-equitable relationships [45].

We expected that our study population might have been different from the general public in terms of attitudes towards equality because of the church influence. Compared with DRC-IMAGES, our findings suggest that their attitudes were not so divergent. Support for gender inequitable norms by church youths might also be due to biased interpretations of some narrow Christian views about patriarchy, which tend to give more power to men, including the control of women’s bodies and sexuality [26, 46]. In certain churches, women are taught to keep silent about violence in relationships since the men deserve “respect” and anything that may lead men to lose that respect should not be exposed to others [47]. Therefore, understanding these gender perspectives may encourage the church to provide more targeted and focused teaching since gender equality is a fairly new topic in Congolese churches [30].

### Gender differences in GEM scale scores

Our findings suggest that women endorsed most of the GEM inequitable statements, indicating that they may experience constant unequal power relationships and therefore internalized inequitable gender norms [28]. The DRC-IMAGES findings stated that women’s responses to the GEM scale may be the result of their lived experiences rather than their true beliefs [28]. Because intimate-partner violence is highly prevalent in DRC [48], women’s coping strategies may include the acceptance of inequitable practices and relationships as survival strategies rather than an option [49]. Our findings are also consistent with other studies examining the relationships of gender attitudes to women’s autonomy [50], and to sexual and reproductive health [51].

A study conducted in Zambia suggested that women’s attitudes and practices around gender norms might partly stem from interpretations of the biblical story of creation as evidence of women’s inferiority in relation to men in relationships [45]. Also, both women and men might hold the view that women are not equal to men [52]. To this end, certain church teachings revolve around women serving men’s sexual needs and subordinating their own sexual feelings to that of men even when they have little benefit. This may exclude women from their own decision making concerning sexual activities [53]. There is a need for the church to develop a sound theologically based-education that can address and challenge interpretations of biblical scriptures leading to gender inequality [54].

### Correlates of the GEM scale

The level of education attained by women and men, especially those with a secondary education or higher, emerged as a consistent predictor of more equitable attitudes and is similar to that of the Mali-IMAGES study [3]. These two study findings suggest that educated people may have progressive views about gender equality and may be aware of its importance [55]. Therefore, educating young women and men about gender equality early in life can prove to be beneficial for increased gender equity when they enter into relationships [56]*.* If educated youth can internalise equitable gender norms and act on them in their daily lives, then a shift in generational gender norms can gradually take place in Africa.

We found that single women and men achieved high GEM scale scores, suggesting that those who may not have experienced a long term serious relationship might have not been exposed to unequal power relationships. And in turn, this might help explain why they have higher GEM scores. From this perspective, research has suggested that certain marriages might be seen as spaces surrounding harmful norms of masculinities, which may reinforce patriarchal attitudes that make it difficult to negotiate recent societal changes, such as gender equality [57, 58]. Our study found a strong positive association between having supportive attitudes towards gender equality and a high GEM scale score in both women and men. This finding is consistent with a study that used the GEM scale to examine the effect on men’s involvement in family planning and HIV services in Uganda [37]. The study findings suggested men’s improvements in some health-seeking behaviours and practices, which included visiting health facilities, taking HIV tests, and using condoms; these behaviours were reported as positively affecting the health of both women and men [37]. The IMAGES findings showed that women who reported that their male partners exhibited more gender equitable behaviours were generally more satisfied with their relationship with their male partners [38].

## Methodological considerations

Our study has some limitations that should be considered when interpreting the results. The relative small sample size could not have identified some relationships by using logistic regression, and a potential selection bias might have been operating because respondents were selected by parish pastors. Because the participants mainly came from the Salvation Army and from specific disadvantaged areas of Kinshasa, the findings might not be generalised to other urban or rural churches of the country. As with any survey carried out on sensitive issues regarding gender equity and sexuality and despite the promise of participant anonymity, some sensitive issues might have been under- and/or over-reported. Although the GEM scale was initially constructed for men, it was applied to both women and men participating in this study to make the findings comparable [59].

## Conclusion

Although most research on gender equity attitudes focuses on men, our study findings suggest that both young women and men had high levels of gender inequitable norms. Hence, our study may have acted as a spotlight, revealing many harmful gender norms that may prevail in the churches and in the society at large. For instance, more women than men were likely to agree on these inequitable attitudes, which highlights how gender inequality is ingrained in both genders and hampers progress of equality. Therefore, churches need to work with both women and men to challenge expectations and harmful norms regarding femininities and masculinities. Overall, the reason why women supported most of the inequitable GEM statements requires further investigation. There is an opening for churches to join hands and use their positions as social institutions that establish and enforce social norms to respond to this challenge.

Women and men with more education, single, and with supportive attitudes for more gender equality had high GEM scale scores. The association between higher educational levels and equitable norms seems to reinforce the importance of education. The church can encourage education, assist youths who have to leave school for income-generating activities, and provide different educational opportunities, especially those that integrate promotion of gender equity. The churches also need to reach young women and men with messages and role models that promote healthy, non-violent and gender equitable lifestyles among church youths.

### Implications for practice

Our study findings indicate the importance of the churches in developing policies and programmes that address gender inequality with particular focus on young people.

1. Churches need to acknowledge the critical responsibilities that women and men can have as key partners in strengthening the response to gender equality and ensure that church staffs, policies and programmes seek to facilitate and advocate for their meaningful involvement.
2. Churches should create, support, and reinforce gender equitable norms, as well as foster alliances with other stakeholders working towards a more gender-equitable future.
3. The focus of church-youth interventions can be broadened to achieve gender-equitable attitudes and practices not just at the individual and family levels but to exercise a wider influence also on community and institutional levels across DRC.

## Appendix

**Table 4:** Attitudes towards gender equality related to relationships between men and women among church-going young women and men in Kinshasa, DRC

Statements	Completely agree		Partly agree		Partly disagree		Disagree	
	Women	Men	Women	Men	Women	Men	Women	Men
	*N* (%)	*N* (%)	*N* (%)	*N* (%)	*N* (%)	*N* (%)	*N* (%)	*N* (%)
When women work they are taking jobs away from men	66 (22.68)	109 (37.72)	49 (19.92)	57 (19.72)	29 (11.79)	25 (8.65)	102 (41.46)	61 (21.11)
When women get rights, they are taking rights way from men	53 (21.29)	91 (31.49)	43 (17.27)	71 (24.57)	37 (14.86)	42 (14.53)	116 (46.59)	55 (19.03)
Rights for women mean that men lose out	26 (10.97)	51 (17.65)	29 (12.24)	41 (14.19)	34 (14.35)	44 (15.22)	148 (62.45)	101 (34.95)
When a woman is raped, she usually did something careless that put herself in that situation	75 (27.47)	91 (31.49)	34 (12.45)	71 (24.75)	43 (15.75)	52 (17.99)	121 (44.32)	47 (16.26)
In some cases, women usually want it to happen	34 (14.05)	67 (23.18)	27 (11.16)	80 (27.68)	25 (10.33)	38 (13.15)	156 (64.46)	73 (25.36)
If a woman did not fight back, you can’t really say it was rape	61 (23.74)	117 (40.48)	57 (22.18)	77 (26.47)	45 (17.51)	31 (10.73)	94 (36.58)	46 (15.92)
In any case, one would have to question whether the “victim” is promiscuous	100 (37.59)	122 (42.26)	40 (15.04)	51 (17.65)	29 (10.90)	39 (13.49)	97 (36.58)	57 (15.92)
In any case, one would have to question whether the “victim” has a bad reputation	83 (29.75)	88 (30.45)	47 (16.85)	78 (26.99)	34 (12.19)	44 (15.22)	115 (41.22)	58 (20.87)

## Abbreviations

AOR: Adjusted odds ratio
CI: Confidence intervals
DRC: Democratic Republic of Congo
EHAIA: Ecumenical HIV and AIDS Initiative and Advocacy
GEM: Gender-equitable men
HIV: Human Immunodeficiency Virus
IMAGES: International Men and Gender Equality Survey
OR: Odds ratios
STI: Sexually Transmitted Infections
UN: United Nations
Vs: Versus
